# On-farm management and participatory evaluation of pigeonpea (*Cajanus cajan* [L.] Millspaugh) diversity across the agro-ecological zones of the Republic of Benin

**DOI:** 10.1186/s13002-020-00378-0

**Published:** 2020-05-13

**Authors:** Géofroy Kinhoégbè, Gustave Djèdatin, Laura Estelle Yêyinou Loko, Abraham Gnimansou Favi, Aristide Adomou, Clément Agbangla, Alexandre Dansi

**Affiliations:** 1 University of Abomey-Calavi, Abomey-Calavi, 01BP526 Benin; 2 BIOGENOM Laboratory, Faculty of Sciences and Technology of Dassa (FAST-Dassa), National University of Sciences Technologies Engineering and Mathematics of Abomey (UNSTIM), BP 14, Dassa-Zoumé, Benin; 3 Laboratory of Applied Entomology, FAST-Dassa, UNSTIM, BP 14, Dassa-Zoumé, Benin; 4 Laboratory of Molecular Genetics and Genomes Analysis, University of Abomey-Calavi, Abomey-Calavi, 01BP526 Benin; 5 Laboratory of Biotechnology, Genetic Resources and Plant and Animal Breeding (BIORAVE), Faculty of Sciences and Technology of Dassa (FAST-Dassa), National University of Sciences Technologies Engineering and Mathematics of Abomey (UNSTIM), BP 14, Dassa-Zoumé, Benin

**Keywords:** Benin, On-farm management, Participatory evaluation, Pigeonpea, Preference criteria, Production constraints

## Abstract

**Background:**

Pigeonpea is a multipurpose food legume crop that contributes to food security in the Republic of Benin. For the establishment of conservation and breeding programs, previous ethnobotanical surveys on pigeonpea were done in Benin but restricted to south and central regions. In previous years, pigeonpea landraces were introduced in northern Benin for soil fertility management; it is therefore important to evaluate the diversity in this legume in this region. Exhaustive documentation of pigeonpea diversity grown in the Republic of Benin will be necessary for effective breeding and conservation programs. Therefore, the aim of this study was to document genetic diversity of pigeonpea, across the agro-ecological zones of the Republic of Benin for its promotion and valorization.

**Methods:**

A total of 500 pigeonpea farmers representing 13 sociolinguistic groups were selected from 50 villages. The data were collected using methods and tools of participatory research appraisal. Folk nomenclatures, taxonomy of pigeonpea and seed system were investigated. The distribution and extent of pigeonpea landraces were evaluated using the Four Square Analysis method. A comparative analysis of pigeonpea use categories production systems, production constraints, famers’ preference criteria, and participative evaluation for existing landraces across agro-ecological zones was done.

**Result:**

Folk nomenclature and taxonomy were mainly based on seed coat color and size. Seven pigeonpea use categories were recorded including sacrifice, grain processing and fertilization. The results showed that the pigeonpea seed system is informal. Based on seed characteristics, fifteen landraces were recorded including seven new landraces. The Sudano-Guinean zone contained the highest number (11) of landraces. The average number of landraces per village was 2.7. A high rate of landraces facing threat of disappearance was observed across the ecological zones. Ten constraints are known to affect pigeonpea production in Benin, with pests and diseases as the most critical in all agro-ecological zones. This study revealed that pigeonpea cultivation is increasing in the Sudanian zone. Varieties to be produced must be selected based on 11 criteria which included precocity and resistance to pests and diseases in the three ecological zones and adaptability to any type of soil in the Sudanian zone. The participatory evaluation revealed the existence of a few performing cultivars.

**Conclusions:**

Our results revealed that implementation of a pigeonpea genetic conservation program in Benin must take into account the diversity, production constraints and varietal preference, which varied according to agro-ecological zones. In situ and ex situ conservation strategies are important to preserve pigeonpea landraces. Morphological and molecular characterizations of identified cultivars are highly recommended to help select suitable varieties for breeding programs.

## Background

Pigeonpea (*Cajanus cajan* [L.] Millspaugh) is a multipurpose food legume, serving as a lifeline to resource-poor farmers in tropical and subtropical regions of Asia, Africa, and Latin America [1]. Pigeonpea is an excellent source of protein (21.7 g/100 g), dietary fibers (15.5 g/100 g), soluble vitamins, minerals, and essential amino acids [2, 3]. Moreover, it is also used in traditional medicines, as its leaves, flowers, roots, and seeds are used for the cure of bronchitis, sores, and respiratory ailments. It also acts as an alexeritic, anthelmintic, expectorant, sedative, and vulnerary [3, 4].

In Benin, pigeonpea is widely consumed in the South-East by the Adja cultural area and contributes to the increase of household incomes [5]. The plant is used for soil conservation and weed management in the fields [5–7]. Despite the importance of pigeonpea [5], very few research efforts have been undertaken to improve the production of the species. As a result, the potential yield of pigeonpea is estimated at 2500 kg/ha, while the yields obtained on farmers’ fields is estimated at 620 kg/ha in Benin [8]. This low yield could be due to the lack of improved varieties in Beninese agriculture [9]. Therefore, an exhaustive collection of cultivated pigeonpea diversity at the country level is the basis for the development of any varietal improvement program and the implementation of conservation strategies.

Several studies have been conducted on pigeonpea diversity in Benin. However, all previous investigations on pigeonpea in Benin were restricted to South and Central Benin [9–12]. These studies reported 7 [11] and 8 [9] pigeonpea landraces with significant differences of diversity across the socio-linguistic groups and decreased production of this legume in Benin [11]. However, there is no data related to pigeonpea diversity and its production constraints in northern Benin although, pigeonpea landraces were introduced in this region for soil fertility management during recent years [13]. In addition, no comparative study on pigeonpea production constraints across different ecological zones in Benin has been documented; varietal diversity as well as farmers’ varietal preference criteria and their variation throughout ecological zones and sociolinguistic groups have very little documentation. While it is known that understanding the genetic diversity, uses, and distribution of orphan crops is essential in determining what to conserve and where to conserve, for sustainable utilization [14–16], it is also important to have a comprehensive collection of pigeonpea genetic resources of the Republic of Benin and to record all associated ethnobotanical knowledge by extensive survey [9, 11].

Seeds are the lifeblood and foundation of successful farming and a crucial element in the lives of agricultural communities [15]. The procedures whereby a cultivar is bred, produced, certified, stored, marketed, and used, which includes all the channels through which farmers acquire genetic materials and the interaction with the commercial seed industry, is known as a seed system [16]. Therefore, the success of introduced crop varieties is tightly linked to the uses, biophysical conditions and the cropping systems in which the crop is integrated which vary across growing areas [10]. Folk taxonomy is a pre-scientific type of naming and classification system rooted in culture [17]. Thus, folk taxonomy is specific to each culture. As a result, vernacular names have a very local distribution and may change with time because of incidental events and contact with other languages [18]. However, folk taxonomy or traditional classification of crop landraces is essential, as these are the basic units that farmers manage, to select and diversify their crops [11]. The knowledge of folk nomenclature and taxonomy is essential for communicating pigeonpea usage in local communities. Unfortunately, pigeonpea folk taxonomy and nomenclature in Benin is restricted [9, 11]. This information is however vital, among others, for developing seed distribution and establishment of regional varietal map [19].

In developing countries where agriculture is the spearhead of the economy, improved varieties must be developed or simply discovered within the existing diversity. In both cases, a good knowledge of the existing varietal diversity and the agronomic performance of varieties are necessary [20, 21]. Hence, farmers’ participation in the varietal selection process determines variety adoption [22]. Moreover, documentation and identification of high-performing cultivars based on farmers’ varietal preference criteria will provide strategies to overcome constraints affecting pigeonpea production in Benin. Consequently, it is important to evaluate the performance of existing pigeonpea landraces under participatory approaches to enhance pigeonpea production and productivity, thereby contributing to the attainment of food security and poverty reduction.

Therefore, this study has been designed with following aims: (1) to document different landraces, local nomenclature and folk taxonomy of pigeonpea grown in Beninese agriculture, (2) to compare seed management and conservation systems of pigeonpea genetic resources and use categories across different ecological zones, (3) to compare constraints associated with pigeonpea production and varietal preference criteria across different ecological zones and sociolinguistic groups, and (4) to evaluate using participatory approaches the performance of different landraces in relation to agronomic and culinary traits.

## Methods

### Study area

**Fig. 1:**
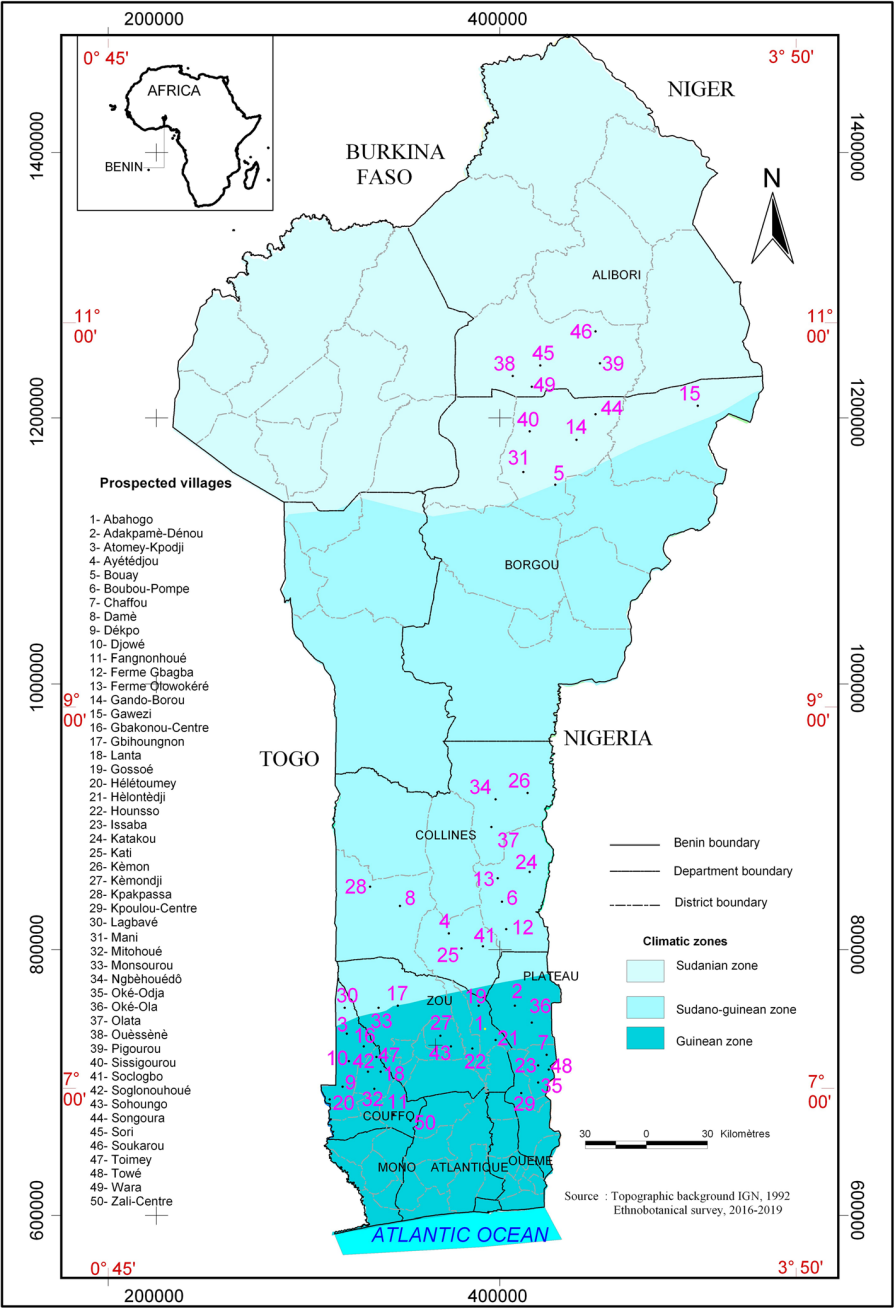
Map of Benin showing the geographical locations of the surveyed villages

The study was carried out in Benin. With a population of 10,008,749 [23], Benin is located in the intertropical zone between parallels 6°30′ North and 12°30′ North latitude, and meridians 1° East and 30°40′ East longitude [24]. With an area of 114,763 km^2^, Benin is bordered in the north by the Niger River in the northwest by Burkina Faso, in the west by Togo, in the south by the Atlantic Ocean and in the east by the Nigeria (Fig. 1). The Republic of Benin is divided into three ecological zones: the Guinean zone (6°25′ North latitude and 7°30′ North longitude) in the South, the Sudano-Guinean zone (7°30′ North latitude and 9°45′ North longitude) in the Central, and the Sudanian zone (9°45′ North latitude and 12°25 North longitude) in the north [25]. The Guinean and Sudano-Guinean zones are both located in a moist agro ecological zone characterized by a subequatorial bimodal climate with two dry seasons and two rainy seasons. The Guinean zone is characterized by an annual rainfall varying between 1200 and 1500 mm/year. The temperature ranges from 24 to 30 °C. The Sudano-Guinean zone annual rainfall varies from 1100 to 1300 mm/year (Table 1). The temperature in this zone varies between 25 and 34 °C. The Sudanian zone is located in the semi-moist agro ecological zone characterized by a unimodal climate pattern with one rainy season and one dry season. The annual rainfall varies between 900 and 1100 mm/year while the temperature ranges from 21 to 35 °C [25] (Table 1).

**Table 1:** Basic information regarding the biophysical characteristics of the surveyed zones

Variables	Guinean zone	Sudano-Guinean zone	Sudanian zone
Altitude (in m)	56–223	153–308	214–609
Annual rainfall (mm)	1200–1500	1100–1300	900–1100
Temperature (°C)	24–30	25–34	21–35
Seasons	Bimodal rainfall regime: 2 dry seasons and 2 rainy seasons	Bimodal rainfall regime: 2 dry seasons and 2 rainy seasons	Unimodal rainfall regime: 1 dry season and 1 rainy season
Dominant soils	Ferrallitic soils	Ferruginous and ferrallitic soils	Concreted or hardened ferruginous soils with small deep
Number of surveyed villages	20	19	11
Surveyed sociolinguistic groups	Biali (Exotic), Somba (Exotic), Fon, Holly, Mahi, Nago, Idaasha	Adja, Fon, Holly, Mahi, Nago, Yoruba	Bariba, Dendi, Peuhl, Yoruba (Exotic), Boo

After an exploratory study in agricultural research institutions, visits to local and urban markets, discussion with farmers and sellers, surveyed villages were selected based on their pigeonpea production, accessibility, and the ability to cover the maximum sociolinguistic groups. A total of 50 villages were selected and included in the survey (Fig. 1).

### Data collection

Surveys were conducted using different methods (group discussions, individual interviews, and field visits) and tools (questionnaires) of participatory research appraisal following Dansi et al*.* [26].

### Focus groups

In each village, groups of 15 to 28 farmers were identified and brought together with the help of administrative and/or local authorities (village chief, farmers’ associations, etc.). Interviews were conducted using local translators to facilitate discussions [27]. Prior to the meetings, farmers were asked to bring samples of pigeonpea landraces they cultivate or knew about. After a brief presentation of the research objectives to the farmers, they were asked to list in vernacular, the names of all pigeonpea landraces cultivated in the village. The distribution and extent of these landraces were evaluated using the participatory method of Four Square Analysis described by Brush [28]. This method allows classifying existing landraces into four groups (produced by many households on large areas, produced by many households on small areas, produced by small households on large areas and produced by few households on small areas). We agreed with the farmers that a landrace cultivated by few households is one grown by no more than 20% of farmers in the context of the village; and a landrace cultivated on a small area is one cultivated on no more than 0.25 ha. The participatory evaluation of identified landraces for agronomic and culinary traits was carried out according to Gbaguidi et al. [29]. The parameters considered were productivity, vegetative cycle, cooking time, sensitivity to pests and disease, and sensitivity to storage insects. The two-level evaluation method described by Loko et al. [30] was used. In this approach, for a given trait, a landrace is scored 1 when it shows good performance and 0 when it shows bad performance. After that, local nomenclature, folk taxonomy, and the vegetative cycle of landraces were documented.

According to Dansi et al. [31], farmers were asked to list all the constraints associated with pigeonpea production. These constraints were prioritized in groups by identifying and gradually eliminating the most severe constraint. As a first step, farmers were asked to identify, among the constraints they have listed, the most critical. The identified constraint is ranked first and eliminated from the list. The same procedure is repeated until the last constraint was ranked. Secondly, farmers were asked to list all the traits that could interest and motivate them to continue growing pigeonpea. Using the same approach (gradual elimination of the most important criterion), the identified criteria were then prioritized. The discussions were free, open-ended, and without a set time limit.

### Household surveys

After the group discussions, 10 households in each selected village were identified for individual interviews. In each household, the person interviewed was chosen based on common agreement from the host couple according to Christinck et al*.* [32]. A total of 500 pigeonpea producers were surveyed throughout the study area. Socio-demographic characteristics (gender, educational level, age, experience, household size), biophysical resources (cropping area, source of labour), cultural practices (sowing period, plant types, land fertilization, pest and disease management, farming activities), and seed system (number of cultivated landrace, sowing time, plant type, land type, perception about the evolution of pigeonpea cultivation, fertilization, sources of labor, level of intervention in the production chain, pests and diseases incidence, and its management and pigeonpea cropping areas for 2015, 2016, and 2017) were recorded. The reasons for pigeonpea production, the different pigeonpea use categories, pest incidence, and its management were also documented. According to their incidence pattern, pest incidence was categorized by farmers as negligible (none), low, average, high, and very high. Incidence was categorized as negligible when pests appeared in very low number while it was categorized as low when infestation was responsible for growth retardation and high when infestation involves damage to flowers or pods and very high when infestation are responsible for death of plant.

### Data analysis

Descriptive statistic was used to analyze data. To avoid overestimation of pigeonpea diversity in each ecological zone, connections between vernacular names were made based on the seed characteristics (seed color, color pattern, pigmentation color, and the seed eye color) according to Mohammed et al. [33], Ayenan et al. [9], and Zavinon et al. [11]. The frequency of disappearance (FD) of each landrace was calculated using the following formula:

$$ \mathrm{FD}=\left(\mathrm{z}/\mathrm{Z}\right)\ast 100 $$

Where *z* = number of landraces threatened to disappear (cultivated by few households on a small areas) and *Z* = total number of landraces identified in the specific ecological zone.

In order to facilitate comparisons and to reduce outliers, from one ecological zone to another, reasons for pigeonpea cultivation and different uses (seed or other plant parts) were categorized [34, 35]. The fidelity level of each category of reason and use was calculated on the scale of each ecological zone according to Akohoué et al. [14]. The fidelity level (FL) was calculated according to the formula described by Friedman [36]:

$$ \mathrm{F}\mathrm{L}=\left(\mathrm{F}/\Sigma \mathrm{F}\right)\ast 100 $$

Where *F* = number of respondents for a given modality of use or reason that motivates pigeonpea cultivation and ΣF = sum of the number of respondents for all modalities of use or reason.

The constraints were prioritized at the level of each ecological zone and the overall study area on the basis of the average of the following three parameters: the total number of villages in which the constraint is cited (TNV), the number of villages where the constraint is the major one or ranked first (MAC), and the number of villages in which the constraint was classified among the principal constraints (PCO), i.e., among the first five. For each of these parameters, a high value indicates an importance for the constraint. Thus, the importance of the constraint (IMC) is determined by the formula described by Dansi et al. [30]:

$$ \mathrm{IMC}=\left(\mathrm{NTV}+\mathrm{MAC}+\mathrm{PCO}\right)/3 $$

The same approach was used to rank farmers varietal preference criteria. To compare the data (socio-demographic characteristics, biophysical resources, cultural practices, and seed system) reported in percentage of responses or in average, analysis of variance (ANOVA), and Tukey test was used for quantitative variables using Minitab 16 Software while the bilateral *Z* test was used for qualitative variables using Statistica 7.1 Software.

For an incidence modality of pests incidence on pigeonpea yield percentage of responses was compared from one ecological zone to another by using bilateral *Z* test. In order to determine potential significant changes in the cropping area from 2015 to 2017, analysis of variance was conducted at the scale of the study area and within each ecological zone. Before ANOVA, data were log-transformed (log(*x* + 1)) for variances homogeneity.

## Results

### Socio demographic characteristics of respondents

In total, 500 pigeonpea producing households including 190 in the Guinean zone, 200 in Sudano-Guinean zone, and 110 in the Sudanian zone were surveyed. Pigeonpea farmers participated in the surveys were from 21 to 76 years old with an average of 45.9 ± 9.2 years old. The majority (62.4%) of pigeonpea farmers were men (62.4%) and illiterate (43.4%), while 31.6% and 25% were found to have primary and secondary levels of education, respectively. The average household size was 6.4 ± 2.1 members (ranging from 3 to 11 members). The years of experience was 15 ± 8 years, on average (Table 2).

**Table 2:** Socio-demographic characteristics of the surveyed pigeonpea producers across agro-ecological zones of Benin

Variables	GZ (*n* = 190)	SGZ (*n* = 200)	SZ (*n* = 110)	Overall (*n* = 500)	Diff
Gender (%)					
Male	56.9^a^	66^a^	65.5^a^	62.4	ns
Female	41.2^a^	44^a^	34.6^a^	37.6	
Education level (%)					
None	56.9^a^	39^b^	28.2^b^	43.4	***
Primary	27.9^a^	34^a^	33.6^a^	31.6	ns
Secondary	15.3^c^	27^b^	38.2^a^	25	***
Age (years)					
Average	48.7^a^	44^b^	44.5^b^	45.9	***
Range	30-69	21-76	26-65	21-76	
Experience (years)					
Average	18.4^a^	16.5^b^	6^c^	15	***
Range	10-45	10-50	3-7	3-50	
Household size (units)					
Average	6.6^a^	6.1^a^	6.4^a^	6.4	ns
Range	3-11	3-11	4-10	3-11	

*GZ* Guinean zone, *SGZ* Sudano-Guinean Zone, *SZ* Sudanean Zone, for the same variable, averages that do not have common letters are statistically different (*p* < 0.05), *ns* non-significant difference at the 5% level

***p < 0.001

Significant differences in surveyed pigeonpea farmers’ ages were observed even across ecological zones. On average, farmers in the Guinean zone are older (48.7 years against 44 years) and more experienced than Sudano-Guinean zone farmers (18.4 years of experience against 16.5). The number of farmers with no education, primary and secondary level of education varied between ecological zones.

### Local nomenclature

Across the thirteen sociolinguistic groups surveyed, 50 different pigeonpea local names were recorded in the local dialects. In reference to the various vernacular names identified, the generic name of pigeonpea varied according to sociolinguistic group and ecological zones (Table 3).

**Table 3:** Pigeonpea generic names variation across sociolinguistic groups

Sociolinguistic groups	Designations	Meanings
Adja	Ekloui, Kloui	Common name
Bariba, Peulh	Wotiri	Pod-producing erected tree
Biali	Tissi Tounan	Referring to cowpea
Boo	Blacia	Referring to cowpea
Fon, Mahi	Hounkoun, Kloué, Klouékoun	Referring to cowpea
Holly, Yoruba	Otini	Common name
Idaasha	Colo	Common name
Nago, Dendi	Otili	Pod-producing tree
Somba	Itoun	Referring to cowpea

In the Guinean and Sudano-Guinean zones, pigeonpea is called Hounkoun, Kloué, or Klouékoun referring to cowpea, by farmers belonging to Fon and Mahi sociolinguistic groups while in the Guinean and Sudanian zones, pigeonpea is called, Otili in reference to a pod-producing tree, by farmers belonging to Nago and Dendi sociolinguistic groups. However, Bariba and Peulh sociolinguistic groups designated pigeonpea by Wotiri in reference to a pod-producing erected tree. Moreover, in the Guinean zone, farmers belonging to Holly and Yoruba sociolinguistic groups designated pigeonpea by Otini. Pigeonpea is called Ekloui or Kloui by Adja sociolinguistic group. In Sudano-Guinean zone, pigeonpea is called Colo (meaning is unknown to farmers) by Idaasha sociolinguistic group while pigeonpea is called Tissi Tounan and Itoun by Biali and Somba sociolinguistic groups respectively, referring to cowpea.

### Folk taxonomy

In the study area, farmers were using 5 different criteria to designate pigeonpea. The great majority of names (90.7%) given to pigeonpea have a meaning. More than half of pigeonpea vernacular names correspond to the morphological aspect (71%) of seeds. This includes seed coat color (85.5%), seed coat and eyes color (9.2%), seed size (1.3%), and seed coat color and size (4%). Plant type (3.7%), seed origin (8.4%), vegetative cycle (10.3%), and in reference to cowpea (3.7%) were also among criteria used by farmers to name pigeonpea (Table 4).

**Table 4:** Meaning of pigeonpea vernacular names across study area

Criteria of denomination	% of vernacular names	Vernacular names	Meaning of the vernacular name
Morphological aspect (seed coat color and size, eyes color)	71	Colo founfoun, Ekloui koudji, Ekloui ri, Hounkoun wéwé, Klouékoun wéwé, Klouékoun wéwé tété Otili founfoun, Otini founfoun, Wotiri gbika, Wotiri goukorou Ekloui djou, Otini dudu	Cream pigeonpea
		Colo kpikpa, Hounkoun vôvô, Klouékoun vôvô, Otili kpoukpa, Otini kpoukpa, Wotiri souan	Red pigeonpea
		Egblèjin, Ekloui wlanwlan, Klouékoun wlanwlan, Otini tchofiti, Wotiri wonka	Mottled pigeonpea
		Klouékoun wéwé noukoun vôvô,	Cream pigeonpea with red eyes
		Klouékoun wéwé noukoun wiwi	Cream pigeonpea with black eyes
		Otili founfoun lakoun,	High sized cream pigeonpea
		Otili founfoun kékélé	Small sized cream pigeonpea
		Ekloui wliwlito	Small sized pigeonpea
Seed origin	8.4	Adja klouékoun, Adja kloui	Pigeonpea from Adja
		CA monlikoun	Pigeonpea introduced by CA
		Carder kloui, Carder kloui	Pigeonpea introduced by Carder
		Djidja kloui	Pigeonpea from Djidja
		Yovo kloui	Pigeonpea introduced by the Europeans
Vegetative cycle	10.3	Bogan	Long vegetative cycle pigeonpea
		Kpèdovinon ovo, Kpèkloué	Short vegetative cycle pigeonpea
		Nontchiovi ekloui, Nontchiovi kloui	Orphan pigeonpea
		Wlétchivé kloui	Pigeonpea producing twice a year
Plant type	3.7	Gbomandoui, Ladja kloui	Tall pigeonpea
		Wotiri	Pod-producing erected tree
Belonging to Cowpea	6.5	Itoun, Tissi tounan, Blacia	Referring to cowpea

The folk taxonomy of pigeonpea has a hierarchical structure with two hierarchy levels as found in several sociolinguistic groups (Adja, Bariba, Fon, Holly, Idaasha, Mahi, Nago, Peuhl, and Yorouba). For example, in the Adja sociolinguistic group, the generic name of pigeonpea Ekloui or Kloui is subdivided into 5 infra-specific pigeonpea taxa (*Ekloui djou, Ekloui koudji*, *Ekloui ri*, *Ekloui wlanwlan*, *Ekloui wliwlito*). In the Fon sociolinguistic group, the generic name of pigeonpea Klouékoun is subdivided into 6 infra-specific pigeonpea taxa (*Klouékoun vôvô*, *Klouékoun wéwé*, *Klouékoun wéwé tété*, *Klouékoun wéwé noukoun vôvô*, *Klouékoun wéwé noukoun wiwi*, *Klouékoun wlanwlan).* While in the Bariba sociolinguistic group, the generic name of pigeonpea Wotiri is subdivided into 4 infra-specific pigeonpea taxa (*Wotiri gbika*, *Wotiri goukorou*, *Wotiri wonka*, *Wotiri souan*).

### Diversity of cultivated pigeonpea landraces

**Fig. 2:**
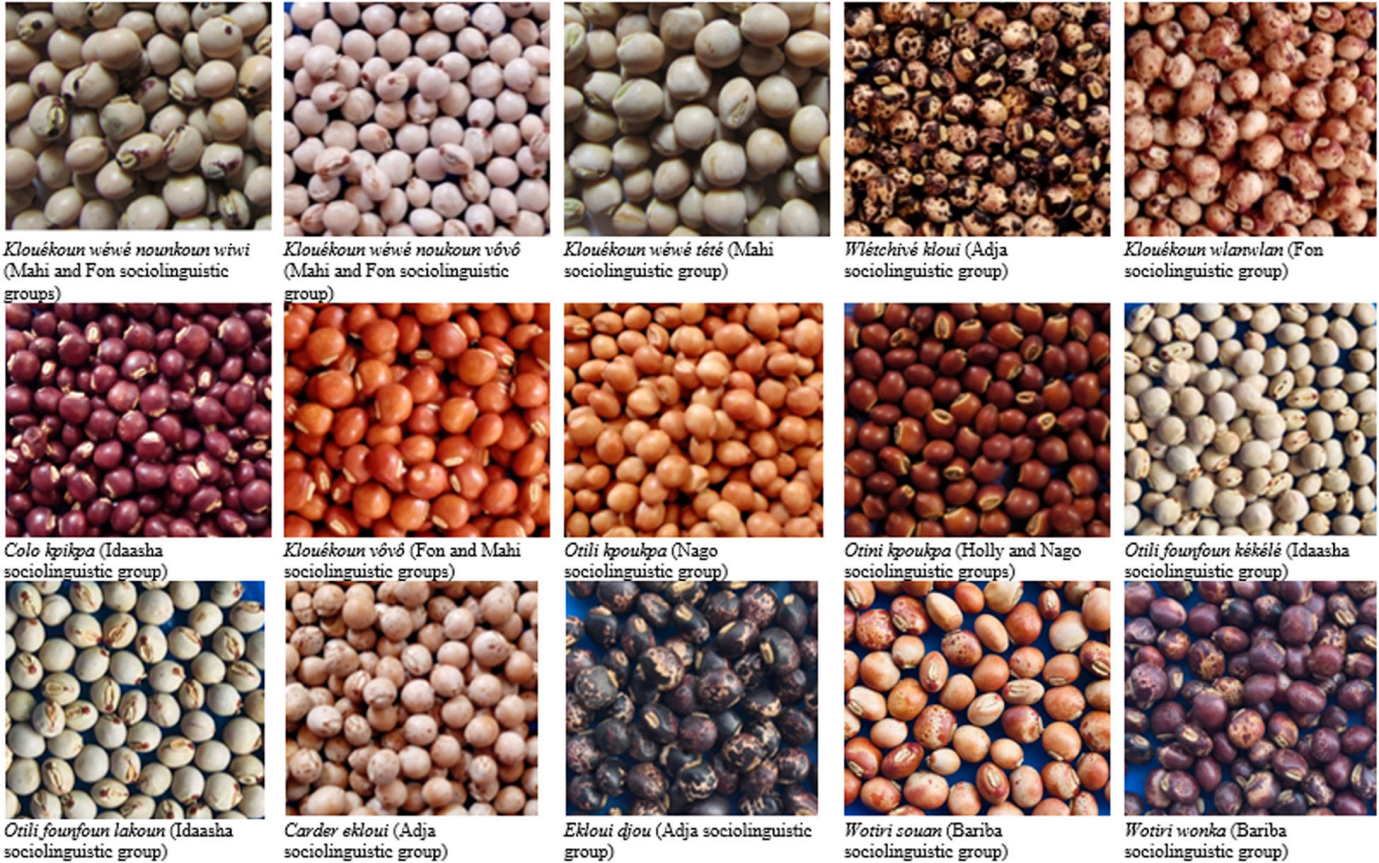
Different pigeonpea landraces cultivated across ecological zones of Benin

Based on seed characteristics, fifteen pigeonpea landraces were identified in the study area (Fig. 2). At village level, the number of pigeonpea landraces ranged from 1 to 5 with an average of 2.7 ± 1. The highest number of landraces (5) per village was reported at Ouèssènè in the department of Alibori. At the household level, the number of pigeonpea landraces held by farmers ranged from 1 to 3 with an average of 1.3 ± 0.5. Specifically, 72.2%, 27.6%, and 0.2% of the farmers cultivated 1, 2, and 3 landraces respectively. The highest number of landraces (3) per household was reported at Ouèssènè in the department of Alibori and maintained by only one farmer. The Sudano-Guinean zone contained the highest number of landraces followed by the Guinean and Sudanian zones (11, 9, and 7 landraces, respectively) while the highest number of landraces (5) per village and per household (3) was recorded in the Sudanian zone.

### Distribution and extent of pigeonpea landraces

Within each ecological zone, pigeonpea production was limited to specific districts and departments. In the Guinean zone, the production was restricted to the districts of Adja-Ouèrè, Kétou and Pobè in the department of Plateau, and the districts of Aplahoué, Klouékanmè, and Lalo in the department of Couffo. In the Sudano-Guinean zone, pigeonpea is cultivated in the districts of Dassa-Zoumè, Ouèssè, Savalou, and Savè in the department of Collines, and the districts of Covè, Djidja, Zakpota, and Zangnannado in the department of Zou. Lastly, in the Sudanian zone, pigeonpea is cultivated in the districts of Bembèrèkè and Kalalé in the department of Borgou, and in the district of Gogounou in the department of Alibori.

The Four Squares Analysis revealed that in the Guinean zone, among the 9 landraces recorded, 1 (*Klouékoun wéwé noukoun vôvô* (Mahi and Fon sociolinguistic groups)) is cultivated by many households on a large area, 1 (*Otili founfoun kékélé* (Idaasha sociolinguistic group)) by few households on large area while the 7 remaining ((*Ekloui djou* (Adja sociolinguistic group), *Wlétchivé kloui* (Adja sociolinguistic group), *Carder ekloui* (Adja sociolinguistic group), *Otili founfoun lakoun* (Idaasha sociolinguistic group), *Klouékoun wéwé tété* (Mahi sociolinguistic group), *Otili kpoukpa* (Nago sociolinguistic group), and *Klouékoun vôvô* (Fon and Mahi sociolinguistic groups)) are produced by few households on a small area.

In the Sudano-Guinean zone, *Klouékoun wéwé nounkoun wiwi* (Mahi and Fon sociolinguistic groups) is cultivated by many households on a large area, *Klouékoun wéwé tété* (Mahi sociolinguistic group) and *Otili founfoun kékélé* (Idaasha sociolinguistic group) are cultivated by few households on a large area, and *Wlétchivé kloui* (Adja sociolinguistic group) and *Klouékoun wlanwlan* (Fon sociolinguistic group) by many households on a small area. *Klouékoun wéwé nounkoun wiwi* (Mahi and Fon sociolinguistic groups), *Otini kpoukpa* (Holly sociolinguistic group), *Colo kpikpa* (Idaasha sociolinguistic group), *Otili founfoun lakoun* (Idaasha sociolinguistic group), *Otili kpoukpa* (Nago sociolinguistic group), and *Klouékoun vôvô* (Fon and Mahi sociolinguistic groups) are cultivated by few households on a small area.

In the Sudanian zone, 2 landraces ((*Klouékoun wéwé nounkoun wiwi* (Mahi and Fon sociolinguistic groups) and *Otili founfoun kékélé* (Idaasha sociolinguistic group)) are cultivated by many households on a large area while 1 landrace ((*Klouékoun wlanwlan* (Fon sociolinguistic group)) is cultivated by few households on a large area. Four landraces ((*Ekloui djou* (Adja sociolinguistic group), *Wotiri wonka* (Bariba sociolinguistic group), *Wotiri souan* (Bariba sociolinguistic group), and *Klouékoun vôvô* (Fon and Mahi sociolinguistic groups)) are cultivated by few households on a small area. Thus, in the Guinean zone, 7 landraces are under threat of disappearance, 6 in the Sudano-Guinean zone versus 4 in the Sudanian zone. In areas where landraces are threatened, the frequency of disappearance varied between 50 and 100% (Table 5).

**Table 5:** Distribution and extent of cultivated pigeonpea landraces across the ecological zones of Benin

Local names	Seeds characteristics	Distribution and extent			FD
		GZ	SGZ	SZ	
*Klouékoun wéwé nounkoun wiwi* (Mahi and Fon sociolinguistic groups)	Cream seed coat with black eye	NA	M−S−^m^	NA	100
*Klouékoun wéwé noukoun vôvô* (Mahi and Fon sociolinguistic groups)	Cream seed coat with red eye and intermediate size	M+S+	M+S+	M+S+	0
*Klouékoun wéwé tété* (Mahi sociolinguistic group)	Cream seed coat	M−S− ^m^	M−S+	NA	50
*Wlétchivé kloui* (Adja sociolinguistic group)	Cream seed coat and highly mottled	M−S− ^m^	M+S−	NA	50
*Klouékoun wlanwlan* (Fon sociolinguistic group)	Cream seed coat and mottled	NA	M+S−	M−S+	0
*Colo kpikpa* (Idaasha sociolinguistic group)	Brown seed coat	NA	M−S− ^m^	NA	100
*Klouékoun vôvô* (Fon and Mahi sociolinguistic groups)	Red seed coat	M−S− ^m^	M−S− ^m^	M-S- ^m^	100
*Otili kpoukpa* (Nago sociolinguistic group)	Light seed coat red	M−S− ^m^	M−S− ^m^	NA	100
*Otini kpoukpa* (Holly sociolinguistic group)	Blackish seed coat	NA	M−S− ^m^	NA	100
*Otili founfoun kékélé* (Idaasha sociolinguistic group)	Cream seed coat with red eye and small size	M−S+	M−S+	M+S+	0
*Otili founfoun lakoun* (Idaasha sociolinguistic group)	Cream seed coat with red eye and high size	M−S− ^m^	M−S− ^m^	NA	100
*Carder ekloui* (Adja sociolinguistic group)	Cream seed coat and mottled with high size	M-S- ^m^	NA	NA	100
*Ekloui djou* (Adja sociolinguistic group)	Black seed coat and mottled	M−S− ^m^	NA	M−S− ^m^	100
*Wotiri souan* (Bariba sociolinguistic group)	Red seed coat and mottled	NA	NA	M−S− ^m^	100
*Wotiri wonka* (Bariba sociolinguistic group)	Purple seed coat and mottled	NA	NA	M−S− ^m^	100
	Total	9/15	11/15	7/15	–

*M+S+* many households on a large areas, *M+S* many households on a small areas, *M−S+* few households on a large areas, *M−S−* few households on a small area, *GZ* Guinean Zone, *SGZ* Sudano-Guinean Zone, *SZ* Sudanian Zone, *FD* frequency of disappearance, *m* landrace threatened to disappear. *FD* = (z/Z)*100 where *z* = number of landraces threatened to disappear (cultivated by few households in a small areas) and *Z* total landrace identified in the ecological zone, *NA* not available

At on-farm level, the landraces distribution analysis revealed that *Otini kpoukpa* (Holly sociolinguistic group), *Colo kpikpa* (Idaasha sociolinguistic group) and *Klouékoun wéwé nounkoun wiwi* (Mahi and Fon sociolinguistic groups) were specific to the Sudano-Guinean zone; *Wotiri souan* (Bariba sociolinguistic group) and *Wotiri wonka* (Bariba sociolinguistic group) specific to the Sudanian zone while *Carder ekloui* (Adja sociolinguistic group) was specific to the Guinean zone. *Klouékoun wéwé nounkoun wiwi* (Mahi and Fon sociolinguistic groups), *Otili founfoun kékélé* (Idaasha sociolinguistic group), and *Klouékoun vôvô* (Fon and Mahi sociolinguistic groups) were cosmopolitan for the three ecological zones. Landraces named *Wlétchivé kloui* (Adja sociolinguistic group), *Otili founfoun lakoun* (Idaasha sociolinguistic group), *Klouékoun wéwé tété* (Mahi sociolinguistic group), and *Otili kpoukpa* (Nago sociolinguistic group) were present in the Guinean and Sudano-Guinean zones, *Ekloui djou* (Adja sociolinguistic group) in the Guinean and Sudanian zones while *Klouékoun wlanwlan* (Fon sociolinguistic group) was present in the Sudano-Guinean and Sudanian zones.

### Reasons for pigeonpea production and use category

Our study has revealed that pigeonpea produced for three main reasons depending on the ecological zones (Table 6). In the Guinean and Sudano-Guinean zones, nutritional value is the main motivation while in the Sudanian zone, the land fertilizing power is the main motivation. The third reason is the market value.

**Table 6:** Reasons for pigeonpea production and use category in Benin

Criteria	Variables	GZ *n* = 190		SGZ *n* = 200		SZ n = 110	
		F	FL	*I* p	FL	I p	FL
Reasons	Market value	107	37.8	80	29.6	66	24.1
	Nutritional value	176	62.2	190	70.4	98	35.8
	Land fertilizing power	–	–	–	–	110	40.2
	ΣF	283	–	270	–	274	–
Use category	Consumption	164	34.8	188	38	96	31.9
	Medicine	92	19.5	75	15.2	72	23.9
	Offering	–	–	7	1.4	–	–
	Sacrifice	–	–	5	1	–	–
	Weed control	15	3.2	19	3.8	23	7.6
	Grain processing	20	4.2	10	2	–	–
	Fertilization	181	38.4	191	38.6	110	36.5
	ΣF	472	–	495	–	301	–

*GZ* Guinean zone, *SGZ* Sudano-Guinean zone, *SZ* Sudanian zone, *n* number of respondents, *FL* fidelity level, *F* number of respondents for a modality of use or reason that motivates the culture, *ΣF* sum of the number of respondents for all modalities of use or reason

The different pigeonpea use categories were mainly concentrated on grains. Based on their fidelity level, pigeonpea is used more in medicine in the Guinean (FL = 19.5%) and Sudanian (FL = 23.9%) zones. According to famers, boiled leaves are administered orally to treat malaria. Also, the decoction of the leaves is used in baths to treat measles and is also used as an antibiotic to treat mouth sores or tooth decay. The roots, when chewed, prevent the rise of snake venom, in the case of snake bite.

The use of pigeonpea grains as an offering for food or symbolic purposes and in sacrifice to divinity to request for more yield the following season is specific to the Sudano-Guinean zone and only restricted to Holly and Nago sociolinguistic groups. While grain processing into donuts is specific to Guinean (FL = 4.3%) and Sudano-Guinean (FL = 2%) zones and only restricted to Holly and Adja sociolinguistic groups. In these zones, pigeonpeas are roasted and ground to flour to sprinkle in sauces as a nutritional supplement by farmers belonging to Holly sociolinguistic group or to make donuts by farmers belonging to Adja sociolinguistic group. Consumption, weeds control, and land fertilization are common to all three ecological zones (Table 6).

### Cultural practices

Pigeonpea was considered as an annual plant by most of the surveyed farmers (93.2%). Only 6.2% of farmers considered this legume as a perennial plant. For the latter, the plant is left in the fields and is harvested the following year. The main pigeonpea farming activities included: ploughing, sowing, weeds control, pod harvest, pod plugging and winnowing. Seeding and weeds control were practiced by all the farmers. Pigeonpea is sown between April and June (73.6%) by intercropping with other seasonal crops (82.8%) or in pure stand (17.2%). Three sources of labor were observed. For farming activities, 13.2% of farmers used family labour, 73% combined family and friends labor while 13.8% used a combination of family, friends and jobber labour (Table 7).

**Table 7:** Biophysical resources, cultural practices and seed system across ecological zones

Variables	GZ (*n* = 190)	SGZ (*n* = 200)	SZ (*n* = 110)	Overall (*n* = 500)	diff
Cropping area in 2015 (ha)					
Average	0.9 ± 0.2^b^	1.3 ± 0.8 ^a^	0.3 ± 0.1^c^	0.9 ± 0.6	***
Range	0.5–1.3	0.5–2.5	0.3–0.5	0.3–2.5	
Cropping area in 2016 (ha)					
Average	0.8 ± 0.1^b^	0.9 ± 0.4^a^	0.4 ± 0.1^c^	0.7 ± 0.3	***
Range	0.5–1	0.5–1.5	0.3–1	0.3–1.5	
Cropping area in 2017 (ha)					
Average	0.5 ± 0.1^b^	0.7 ± 0.3^a^	0.4 ± 0.1^c^	0.6 ± 0.3	***
Range	0.6–0.8	0.5–1.25	0.3–0.8	0.3–1.3	
Source of labor (%)					
Family labor	12.1^b^	13.5^b^	49.3^a^	13.2	***
Family and friends labor	87.9^a^	61.5^b^	42.2^c^	73	***
Family friends and jobber labor	–	25^a^	8.5^b^	13.8	***
Seed system (%)					
Seeds of previous harvests	70^a^	62.9^a^	46.2^b^	60.2	***
Seeds of friends	9.2^b^	10.5^b^	50.4^a^	22	***
Market seeds	20.8^a^	26.7^a^	3.4^b^	17.8	***
Conservation method	3.7^a^	4.5^a^	–	3.2	ns
Seed storage	70^b^	84^a^	34.6^c^	67.8	***
Seed purchase	30^b^	16^c^	65.5^a^	32.2	***
Plant type (%)					
Annual plant	92.1^b^	90.5^b^	100^a^	93.2	***
Perennial plant	7.9^a^	9.5^a^	–	6.8	ns
Land type (%)					
Intercropping system	100^a^	98.5^b^	24.6^c^	82.8	***
Pure stand	–	1.5^b^	75.4^a^	17.2	***
Land, pests and diseases management (%)					
Fertilization	–	–	–	–	ns
Use of pesticides	–	–	63.7	14	***
Sowing period (%)					
April–June	97.9^a^	91^b^	–	73.6	***
April–May	2.1^a^	9^b^	–	4.4	***
June–August	–	–	68.2	15	***
July–August	–	–	31.8	7	***
Farming activities (%)					
Ploughing	65.8^b^	83^a^	72.7^b^	74.2	***
Sowing	100^a^	100^a^	100^a^	100	ns
Weed control	100^a^	100^a^	100a	100	ns
Pod harvest	92.6^b^	97.5^a^	87.3^b^	93.4	***
Pod plugging	84.7^b^	96^a^	80.9^b^	88.4	***
Winnowing	52.1^b^	17^c^	74.5^a^	43	***
Yield (kg/ha)					
Average	557.5 ± 15.9^a^	566.6 ± 35.8^b^	522.3 ± 44^c^	553.4 ±36.3	***
Range	450–560	550–650	450–560	450–650	

*GZ* Guinean zone, *SGZ* Sudano-Guinean Zone, *SZ* Sudanean Zone, *n* number of respondents, *diff* difference, for the same variable, means, or percentages that do not have common letters are statistically different (*p* < 0.05), *ns* non-significant difference at the 5% level

***p < 0.001

The activity of land fertilization was never practiced by farmers included in this study while only 14% of farmers included in this study used pesticide. The average grain yield in farmers’ fields was estimated at 553.4 ± 36.3 kg/ha. According to the farmers, during the three last years, Sudano-Guinean zone was the largest cropping area followed by the Guinean zone while farmers in the Sudanian zone produced pigeonpea on a small cropping area (Table 7). Sowing was actively done between April and June in the Guinean and Sudano-Guinean zones (97.9% and 91% respectively) whereas it was actively done in June, July, and August in the Sudanian zone (68.2%). Intercropping with other seasonal crops such as maize and millet was specific to the Guinean (100%) and Sudano-Guinean (98.5%) zones while pigeonpea was cultivated mostly in pure stand in the Sudanian zone (75.4%).

Family and friends were the main source of labor for various farming activities in the Guinean and Sudano-Guinean zones (87.9% and 61.5% respectively) while family members (49.3%) were the main source of labor in the Sudanian zone. Our results revealed that the average yield of pigeonpea in the Sudanian zone is lower (522.3 ± 44 kg/ha) than that of the Guinean and Sudano-Guinean zones (557.5 ± 15.9 kg/ha and 566.6 ± 35.8 kg/ha, respectively).

### Seed system

Different sources of seeds were mentioned by farmers. Previous harvest (60.2%) is the main source of seeds. Other sources are borrowing of seeds from friends (22%) and seeds purchased from the local market (17.8%). After each harvest, 67.8% of farmers stored seeds until scarcity at market or for the following season while 32.2% of them sold seeds in local markets. The comparison of the seed systems between ecological zones revealed that previous harvest is the main source of seeds in the Guinean and Sudano-Guinean zones (70% and 62.9%, respectively) and borrowing of seeds from friends is the main source (50.4%) in the Sudanian zone. After each harvest, farmers stored more grains in the Guinean and Sudano-Guinean zones (70% and 84%, respectively) while more grain was immediately sold in the Sudanian zone (65.5%) (Table 7).

### Pigeonpea production constraints

In total, 10 constraints were identified as major bottleneck in pigeonpea production. Among which, the long vegetative cycle, pests, diseases, and rainfall irregularity were considered as the major constraints (Table 8). According to the farmers’ descriptions, low productivity ranked seventh among the constraints followed by the sensitivity to storage insects. All these constraints have been reported in the three ecological zones. However, their relative importance varied from one zone to another. The most important constraint in the Guinean and Sudano-Guinean zones was the long vegetative cycle. Sensitivity to pests and diseases ranked second. In the Sudanian zone, pests and diseases ranked as the most important constraint and soil poverty was second (Table 8).

**Table 8:** Comparative table of pigeonpea production constraints across ecological zones

Constraints	Overall					Rank per zone		
	TNV	MAC	PCO	Imp	Rank	GZ	SGZ	SZ
Long vegetative cycle	50	28	48	42	1	1	1	3
Pests and diseases	48	14	48	36.7	2	2	2	1
Rainfall irregularity	30	3	25	19.3	3	3	4	4
Weeding	25	1	23	16.3	5	4	5	6
Lack of improved varieties	26	-	24	16.7	4	5	3	5
Storage insects	10	-	7	5.7	8	6	9	8
Soil poverty	15	3	13	10.3	6	7	8	2
Harvest and post-harvest work	10	1	4	5	9	8	9	7
Low productivity	11	-	10	7	7	9	6	6
Lack of cultivable land	5	-	3	2.7	10	10	7	9

GZ: Guinean zone; SGZ: Sudano-Guinean zone; SZ: Sudanian zone; TNV: Total Number of Villages in which the constraint is cited; MAC: Number of villages where the constraint is the major one or ranked first; PCO: number of villages in which the constraint was classified among the principal constraints i.e. among the first five; Imp: Importance

### Incidence of pests on pigeonpea yield and control methods

The incidence of pests and diseases on farmers’ field was as follows: low in the Guinean and Sudano-Guinean zones (52.6% and 42.5%, respectively), high in the Sudanian zone (81.8%) (Table 9). As a result, farmers reported growth retardation and damage to flowers or pods respectively. A pest control method was only reported in the Sudanian zone (63.7%). Three reasons justified the non-control of pests: high price of pesticides (49.6%), risk of intoxication (29.6%), and lack of sprayers (20.8%).

**Table 9:** Evaluation of pests and diseases impact in pigeonpea production

Evaluation (% of responses)	Variables	Overall (*n* = 500)	GZ (*n* = 190)	SGZ (*n* = 200)	SZ (*n* = 110)	Diff
Impact on pigeonpea yield	None	12.8	18.4^b^	28.5^a^	2.7^c^	***
	Low	51.3	52.6^a^	42.5^b^	1.8^c^	***
	Average	32.1	29^a^	29^a^	9.1^b^	***
	High	2.6	–	–	81.8	***
	Very high	1.3	–	–	4.6	***
Control of pests and diseases	Absence of control		–	–	36.3	***
	Use of cotton pesticides		–	–	63.7	***

*GZ* Guinean zone, *SGZ* Sudano-Guinean zone, *SZ* Sudanian zone, *n* number of respondents; Diff: difference; for the same variable, means or percentages that have no common letters are statistically different (*p* < 0.05), *ns* non-significant difference at the 5% level

***p < 0.001

### Evolution of pigeonpea production in Benin

Overall, majority of the farmers (69.4%) reported a decrease of pigeonpea production in Benin. This downward trend was observed for the Guinean and Sudano-Guinean zones (75.79% and 85.5%, respectively). In these zones, the decrease in cropping area is highly significant (*p* < 0.001). The average cropping area was 0.9 ± 0.2 ha in 2015, 0.8 ± 0.1 ha in 2016, and 0.5 ± 0.1 ha in 2017 in the Guinean zone (Table 7). Similarly, in the Sudano-Guinean zone, average cropping area was 1.3 ± 0.8 ha in 2015, 0.9 ± 0.4 ha in 2016, 0.7 ± 0.3 ha in 2017 (Table 7). In contrast, in the Sudanian zone, pigeonpea cultivation is increasing (70.91%). In this zone, the increase of cropping area is highly significant (*p* < 0.001). The average cropping area was 0.3 ± 0.1 ha in 2015, 0.4 ± 0.1 ha in 2016 and 0.4 ± 0.1 ha in 2017 (Table 7). This increase is due to the fertilizing power of the plant (89.1%) and weed control (10.9%).

### Farmers’ preference criteria of pigeonpea

Throughout the study, 11 criteria depending on the ecological zones and different sociolinguistic groups highlighted the choice of pigeonpea varieties to be cultivated by farmers. Farmers perceived precocity, resistance to pests and diseases, short cooking time, adaptability to any type of soil, good taste, and high productivity as the most important preferred traits (Table 10). In the Guinean and Sudano-Guinean zones, farmers had a strong preference for early maturing (precocity) and resistant to pests and diseases pigeonpea varieties; while in the Sudanian zone, farmers preferred pigeonpea varieties that were resistant to pests and diseases and adaptable to any type of soil (Table 10). Precocity appeared high on the list of criteria for all sociolinguistic groups except Nago sociolinguistic group for whom adaptability to any type of soil was the first criterion. Farmers belonging to the Bariba sociolinguistic group preferred varieties that mature early, are resistant to pests and diseases, have short cooking time, show adaptability to any type of soil and have good taste (Table 11). In addition to Bariba sociolinguistic group’s preferred traits, farmers belonging to Boo sociolinguistic group showed strong tendency towards pigeonpea varieties that are cultivable at any time of the year and resistant to storage insects. Dendi sociolinguistic group preferred varieties with high productivity and cultivable at any time of the year and Peuhl sociolinguistic group preferred highly productive and resistant to storage insects pigeonpea varieties. Precocity, resistance to pests and diseases, short cooking time, and adaptability to any type of soil were farmers belonging to the Yoruba sociolinguistic group preferred traits.

**Table 10:** Farmers’ preference criteria of pigeonpea across ecological zones

Preference criteria	Overall					Rank per zone		
	TNV	MCR	PCr	Imp	Rank	GZ	SGZ	SZ
Precocity	50	23	49	40.7	1	1	1	3
Resistance to pests and diseases	45	16	45	35.3	2	2	2	1
Short cooking time	39	0	30	23	4	3	5	5
Adaptability to any type of soil	28	5	25	19.3	6	4	8	2
Good taste	38	0	35	24.3	3	5	4	4
High productivity	35	3	23	20.3	5	6	3	7
Cultivable at any time of the year	22	1	10	11	7	7	7	8
High market value	11	2	10	7.7	8	8	6	-
Resistance to storage insects	10	0	5	5	9	9	10	6
Easy for ginning	6	0	1	2.3	11	10	–	10
Drought resistance	11	0	3	4.7	10	10	9	9

*GZ* Guinean zone, *SGZ* Sudano-Guinean zone, *SZ* Sudanian zone, *TNV* total number of villages in which the criterion is cited, *MCR* number of villages where the criterion is the major one or ranked first, *PCr* number of villages in which the criterion was classified among the principal criterion, i.e., among the first five, *Imp* importance

**Table 11:** Importance (in rank) of varietal preference criteria across different sociolinguistic groups

Preference criteria	Adja	Bariba	Biali	Boo	Dendi	Fon	Holly	Idaasha	Mahi	Nago	Peuhl	Somba	Yorouba
Precocity	1	1	1	1	1	1	1	1	1	3	1	1	1
Resistance to pests and diseases	2	1	–	1	1	2	1	1	2	3	1	1	1
Short cooking time	2	1	–	1	1	3	1	2	2	2	1	–	1
Adaptability to any type of soil	5	1	1	1	1	5	2	–	5	1	1	–	1
Good taste	4	1	1	1	1	2	1		3	2	1	1	2
High productivity	3	2	1	2	1	2	3	2	2	2	1	–	3
Cultivable at any time of the year	5	3	–	1	1	6	3	2	4	1	2	–	1
High market value	6	–	1	–	–	6	2	1	5	–	–	1	–
Resistance to storage insects	–	3	–	1	–	7	–	–	5	–	1	–	4
Easy for ginning	7	4	–	2	–	–	–	–	5	–	2	–	4
Drought resistance	–	2	–	2	–	4	–	–	–	–	–	–	3

### Participatory evaluation of pigeonpea landrace grown in Benin

Our results revealed that none of the landraces identified simultaneously in the three ecological zones is performing for a given character simultaneously in the three ecological zones (Table 12). Moreover, no landrace is performing simultaneously for all 5 evaluated traits. Nevertheless, *Carder ekloui* (Adja sociolinguistic group) specific to the Guinean zone combined 4 good performances (high productivity, short cooking time, resistance to pests and diseases, and resistance to storage insects). *Carder ekloui* (Adja sociolinguistic group) and *Otili founfoun kékélé* (Idaasha sociolinguistic group) showed high productivity in the Guinean and Sudano-Guinean zones but showed low productivity in the Sudanian zone, however, these two landraces, showed resistance to pests and diseases. *Klouékoun vôvô* (Fon and Mahi sociolinguistic groups) showed high productivity, short cooking time, resistance to pests and diseases, resistance to storage insects, and short vegetative cycle in the Guinean and Sudano-Guinean zone, but showed low productivity and susceptibility to pests and diseases in the Sudanian zone (Table 12).

**Table 12:** Agronomic and culinary characteristics of pigeonpea landraces grown across Benin’s ecological zone

Landraces/local names	GZ	SGZ	SZ
CBSL *Klouékoun wéwé nounkoun wiwi* (Mahi and Fon sociolinguistic groups)	NA	High productivity; long cooking time; long vegetative cycle; Resistant to diseases and pests; Susceptible to storage insects	NA
CRISL *Klouékoun wéwé noukoun vôvô* (Mahi and Fon sociolinguistic groups)	High productivity; long cooking time; long vegetative cycle; resistant to diseases and pests; susceptible to storage insects	High productivity; long cooking time; long vegetative cycle; resistant to diseases and pests; susceptible to storage insects	Low productivity; long cooking time; long vegetative cycle; resistant to diseases and pests; susceptible to storage insects
CSL *Klouékoun wéwé tété* (Mahi sociolinguistic group)	High productivity; long cooking time; long vegetative cycle; resistant to diseases and pests; resistant to storage insects	High productivity; long cooking time; long vegetative cycle; resistant to diseases and pests; resistant to storage insects	NA
CHMSL *Wlétchivé kloui* (Adja sociolinguistic group)	Low productivity; long cooking time; long vegetative cycle; resistant to diseases and pests; resistant to storage insects	Low productivity; long cooking time; long vegetative cycle; resistant to diseases and pests; resistant to storage insects	NA
CMSL *Klouékoun wlanwlan* (Fon sociolinguistic group)	NA	Low productivity; long cooking time; long vegetative cycle; resistant to diseases and pests; resistant to storage insects	Low productivity; long cooking time; long vegetative cycle; susceptible to diseases and pests; resistant to storage insects
BWSL *Colo kpikpa* (Idaasha sociolinguistic group)	NA	Low productivity; long cooking time; long vegetative cycle; resistant to diseases and pests; resistant to storage insects	NA
RSL *Klouékoun vôvô* (Fon and Mahi sociolinguistic groups)	High productivity; short cooking time; short vegetative cycle; resistant to diseases and pests; resistant to storage insects	High productivity; short cooking time; short vegetative cycle; resistant to diseases and pests; resistant to storage insects	Low productivity; short cooking time; short vegetative cycle; Susceptible to diseases and pests; resistant to storage insects
LRSL *Otili kpoukpa* (Nago sociolinguistic group)	High productivity; short cooking time; short vegetative cycle; resistant to diseases and pests; resistant to storage insects	High productivity; short cooking time; short vegetative cycle; resistant to diseases and pests; resistant to storage insects	NA
BKSL *Otini kpoukpa* (Holly sociolinguistic group)	NA	Low productivity; long cooking time; long vegetative cycle; resistant to diseases and pests; resistant to storage insects	NA
CRSSL *Otili founfoun kékélé* (Idaasha sociolinguistic group)	High productivity; long cooking time; long vegetative cycle; resistant to diseases and pests; susceptible to storage insects	High productivity; long cooking time; long vegetative cycle; resistant to diseases and pests; susceptible to storage insects	Low productivity; long cooking time; long vegetative cycle; resistant to diseases and pests; susceptible to storage insects
CRHSL *Otili founfoun lakoun* (Idaasha sociolinguistic group)	High productivity; long cooking time; long vegetative cycle; resistant to diseases and pests; susceptible to storage insects	High productivity; long cooking time; long vegetative cycle; resistant to diseases and pests; susceptible to storage insects	NA
CMHSL *Carder ekloui* (Adja sociolinguistic group)	High productivity; short cooking time; long vegetative cycle; resistant to diseases and pests; resistant to storage insects	NA	NA
BMSL *Ekloui djou* (Adja sociolinguistic group)	High productivity; long cooking time; long vegetative cycle; resistant to diseases and pests; resistant to storage insects	NA	Low productivity; long cooking time; long vegetative cycle; susceptible to diseases and pests; resistant to storage insects
RMSL *Wotiri souan* (Bariba sociolinguistic group)	NA	NA	Low productivity; long cooking time; long vegetative cycle; susceptible to diseases and pests; resistant to storage insects
PMSL *Wotiri wonka* (Bariba sociolinguistic group)	NA	NA	Low productivity; long cooking time; long vegetative cycle; susceptible to diseases and pests; resistant to storage insects

*BKSL* Blackish seeded landrace, *BMSL* black and mottled seeded landrace, *BWSL* brown seeded landrace, *CBSL* cream with black eye seeded landrace, *CHMSL* cream and highly mottled seeded landrace, *CMHSL* cream and mottled with high size seeded landrace, *CMSL* cream and mottled seeded landrace, *CRHSL* cream with red eye and high size seeded landrace, *CRISL* cream with red eye and intermediate size seeded landrace, *CRSSL* cream with red eye and small size seeded landrace, *CSL* cream seeded landrace, *LRSL* light red seeded landrace, *PMSL* purple and mottled seeded landrace, *RMSL* red and mottled seeded landrace, *RSL* red seeded landrace, *GZ* Guinean zone, *SGZ* Sudano-Guinean zone, *SZ* Sudanian zone, *NA* landrace absent

## Discussion

Our study showed that pigeonpea generic names varied according to sociolinguistic group and ecological zones. Our findings are similar to Ayenan et al. [9] and Zavinon et al. [11] in Southern Benin. However, pigeonpea is designated by the same generic name by farmers belonging to different sociolinguistic groups in the same ecological zone or different ecological zones. This convergence in generic names within different sociolinguistic groups could be explained by the fact that these groups could have common origins or cohabitation could have facilitated the transfer of knowledge over time.

In addition, based on the meaning of generic names, famers across the sociolinguistic groups within three ecological zones recognized pigeonpea by referring to it as cowpea. This suggests that famers do not have a good knowledge of the botanical systematic of pigeonpea. There is therefore no link between folk taxonomy and the scientific classification of pigeonpea. Since there could be a connection between folk taxonomy and scientific classification of the species [37], our findings are contrary to Akohoué et al. [14] on Kersting’s groundnut in Benin. However, the hierarchical characterization of pigeonpea folk taxonomy was like the observation made by Loko et al. [38] on common beans and reflects the high diversity level of pigeonpea in the surveyed sociolinguistic groups.

Our results from Adja sociolinguistic group infra-specific pigeonpea taxa was contrary to that of Ayenan et al. [9] and Zavinon et al. [11] who distinguished respectively 2 and 3 infra-specific pigeonpea taxa. However, local names do not necessarily reflect the genetic history of landraces because different names may be given to identical seeds of landraces or a single name may apply to heterogeneous crops [39]. This situation may contribute to under or over-estimation of the diversity within species [9, 25, 40, 41]. To avoid redundancies and optimize the efficient conservation and sustainable use of pigeonpea diversity, it is important to conduct morphological and molecular characterizations to avoid redundancies and establish equivalence between the local names [28, 42, 43].

Farmers used the morphological aspect of seeds (coat color, seed eye color, and seed size), plant type, seeds origin, and vegetative cycle for folk varieties’ identification. These criteria of pigeonpea classification and identification are among the descriptors of *C. cajan* recommended by International Board for Plant Genetic Resources (IBPGR) and International Crops Research Institute for the Semi-Arid Tropics (ICRISAT) [44] and used by many authors in morphological characterization of this legume. Our study revealed that the morphological aspect of seeds (particularly the seed coat color) was the predominant criterion used by the farmers to classify and identify pigeonpea landraces. The main reason is that seed coat color is unique to each landrace while other traits may be commonly shared [14]. However, our findings were contrary to Manyasa et al. [45] who reported pigeonpea seed size and maturity as the most important criteria used by the famers in Ugandan. Although, similar observations were reported by Esan and Ojemola [46] in Nigeria, Ayenan et al. [9] and Zavinon et al. [11] in Southern and Central Benin and suggests that selection based on the morphological aspect of seeds will have a definite role in the framework of on-farm conservation of this legume in Benin.

According to Ayenan et al. [9] and Zavinon et al. [11], eight and seven pigeonpea landraces respectively were recorded in Benin. In our study, we recorded fifteen pigeonpea landraces based on seed characteristics. The seven new landraces are the *blackish seeded landrace* called *Otini kpoukpa* (Holly sociolinguistic group), the *brown seeded landrace* called *Colo kpikpa* (Idaasha sociolinguistic group), the *cream with black eye seeded landrace* called *Klouékoun wéwé nounkoun wiwi* (Mahi and Fon sociolinguistic groups), the *cream seeded landrace* called *Klouékoun wéwé tété* (Mahi sociolinguistic group), the *light red seeded landrace* called *Otili kpoukpa* (Nago sociolinguistic group), the *purple and mottled seeded landrace* called *Wotiri wonka* (Bariba sociolinguistic group), and the *red and mottled seeded landrace* called *Wotiri souan* (Bariba sociolinguistic group). Considering that previous studies did not take into account the entire production area, though seemingly insignificant, a part of the existing pigeonpea landraces in Benin was left out. This finding suggests that the extent of the area studied affects species richness [14, 47]. Thus, a study which aims to reflect the existing diversity of cultivated species should not be restricted to only the major production areas of the species.

Our results revealed that pigeonpea diversity at on-farm level was specific to ecological zones. In fact, each landrace did not have the same distribution and extent across ecological zones. For instance, *Otili founfoun kékélé* (Idaasha sociolinguistic group) was cultivated by a few households on large areas in the Guinean and Sudanian-Guinean zones while it was cultivated by many households on a large area in the Sudanian Zone. This practice involves an indirect selection of some landraces in certain ecological zones while it promotes a gradual disappearance of another. Therefore, conservation strategies should be defined for landraces under threat of disappearance according to their ecological zones. Thus, each ecological zone could be a favorable candidate for in-situ conservation of pigeonpea genetic resources in Benin. The highest number of landraces per village and per household was recorded at Ouèssènè in the department of Alibori in the Sudanian zone. Therefore, the Sudanian zone is essential in the conservation of pigeonpea genetic resources in Benin and confirms that each ecological zone could be a favorable candidate for in-situ conservation of pigeonpea genetic resources as suggested previously.

It did not come as a surprise that the fertilizing power of pigeonpea was recorded as a major reason for producing this legume, because pigeonpea has a significant position in dry land farming systems. This is especially adopted by small and marginal farmers in many parts of the world by fixing nitrogen and flexibility for mixed cropping or inter crop [48, 49]. The use of pigeonpea leaves to treat various diseases such as malaria corroborates the observations made by Ayenan et al. [9] and Zavinon et al. [11] in Benin and those of Aiyeloja and Bello [50] and Oladunmoye et al. [51] in Nigeria. Also, the use of pigeonpea as weed control has been reported by several authors in Benin [5, 52, 53]. Nonetheless, pigeonpea root utilization in prevention of snake venom rising and grain processing into donuts has not been reported by previous studies.

Unfortunately, this technological ability of pigeonpea grains is weakened by its oil retention. Thus, this technological ability of pigeonpea grains must be explored and improved, like the soybean’s transformation into cheese, in Benin. This will reduce malnutrition in rural populations and could contribute to the in-situ conservation of the existing pigeonpea diversity. Moreover, the use of pigeonpea grains as an offering for food or for symbolic purposes and in sacrifice to divinity has not yet been reported by previous research. All these findings are dependent on sociolinguistic groups and ecological zones and suggest that pigeonpea farmers in Benin do not have the same knowledge of pigeonpea uses. However, as a result of vertical knowledge transmission [14, 54], specific knowledge relative to the plant part uses might be kept and transmitted within communities in some areas. With the knowledge that integrating cultural practices of local communities leads to an efficient on-farm conservation [38, 55], this specific use category of pigeonpea genetic resource shows the potential of the cultural approach for the conservation of this legume in Benin.

Our study revealed that in the Sudanian zone, pigeonpea cultivation is increasing while it is decreasing in the Guinean and Sudano-Guinean zones. Our results in the Guinean and Sudano-Guinean zones corroborate Zavinon et al. [11]. In fact, the productivity of the smallholder farming system in the Sudanian zone is under threat due to soil fertility decline [56]. Research in many parts of Africa including Benin have shown that legumes have the potential to sustain soil fertility in smallholder farming systems [49, 57, 58]. Consequently, thanks to the Protection and Rehabilitation of Soils to Improve Food Security project of the German Federal Ministry for Economic Cooperation and Development, an integrated soil fertility management through maximum use of different organic sources of fertilizers such as pigeonpea, was initiated in 2015. This project allowed, in the Sudanian zone, the popularization of pigeonpea by using its fertilizing power and supports the vital role of the Sudanian zone for the in-situ conservation of pigeonpea genetic resources in Benin.

As reported by Ayenan et al. [10] and Zavinon et al. [12], our study showed that pigeonpea seed system in Benin is informal. Similar results were achieved in Tanzania [59] and in India [15]. This informal seed system has the advantage of facilitating seed exchanges among farmers and villages [16]. Nevertheless, marketed seeds deserve some attention taking into consideration that seed acquisition from the market does not guarantee genetic purity [60]. It is extremely important to have good quality seeds available to farmers, in order to increase pigeonpea productivity [10, 60]. At on-farm level, the intercropping system of pigeonpea with other crops has been found in other countries such as Uganda [45] and Kenya [61]. Accordingly, after each harvest, majority of farmers stored seeds until scarcity at market before selling them. On the contrary, farmers in the Sudanian zone sold their seeds immediately to address urgent financial burdens such as educating their children. As a result, pigeonpea is an essential source of household income and reduces poverty in Benin as reported by Dansi et al. [5].

In Benin, there are many factors negatively affecting pigeonpea production. Long vegetative cycle and pests and diseases were the main constraints affecting pigeonpea production. Indeed, African pigeonpea is characterized by its late maturity [12, 62]. According to farmers, these genotype cultivation as a sole crop occupies land which could be used for other crops. Our results were in accordance with Ayenan et al. [10] and Zavinon et al. [12] who showed the lack of improved varieties (long vegetative cycle, low productivity, insect attack and lack of quality seed) as the main constraint affecting pigeonpea production in the Guinean and Sudano-Guinean zones of Benin. Moreover, the occurrence of pests and diseases as the top constraint in the Sudanian zone is hardly surprising. In this zone, pigeonpea was cultivated mostly on pure land which facilitates pests’ attraction. Our results confirmed the observation made by Sarkar et al. [49] that intercropping system minimizes pests and diseases attraction compared to the pure stand system. Farmers in this zone had limited access to pesticides and were suffering the most from production loss. Although the impact of pests and diseases was found to be low in the study area, their presence is a key indicator of the urgent need to develop strategies against these pests. Instead of the use of pesticides, an integrated pests management system is recommended, through the combination of biological control based on use of natural enemies of these pests and genetic control based on use of tolerant or resistant varieties [29, 63, 64].

As is the case with various legumes where storage insects are the major constraint [65], surveyed farmers reported seeds’ attack by storage insects. Farmers reported the use of toxic products to protect their seeds. Thus, an education of farmers or consumers for a purely biological conservation, such as the use of small peppers, is highly recommended, as in the case of Kersting’s groundnut [66]. Curiously, low productivity ranked seventh among the constraints and suggests that low productivity represents only a small portion of the constraints relative to pigeonpea production and could be the direct consequence of the negative effects of other constraints [61]. Therefore, the lack of improved varieties appears as a challenge to pigeonpea production. Hence, the availability of improved varieties and their distribution across the different ecological zones according to their specific needs can alleviate pigeonpea production constraints in Benin. The government should encourage small-scale enterprises to provide farmers with improved seeds.

A farmer’s preference criteria plays an important role in breeding programs and facilitates the adoption of improved varieties [11, 40]. Our study revealed that famers perceived precocity, resistance to pests and diseases, good taste, and short cooking time as the most important preferred traits. Similar observations on pigeonpea were made by Mergeai et al. [61] in Kenya, Shiferaw et al. [67] in Tanzania, Changaya [68] in Malawi, Ogbe and Bamidele [69] in Nigeria, and Ayenan et al. [10] in Southern Benin. All these criteria were correlated with identified constraints. This suggests a veritable link between these two parameters as reported by Odjo et al. [70] on rice genetic resources in Benin. The precocity as criterion is important for famers because short vegetative cycle varieties should certainly encourage them to produce pigeonpea. In the global climate context where changes are noticeable, early varieties will provide producers the guarantee that pigeonpea plants attain a significant level of vegetative development before the rain cuts. The high productivity as criterion of varietal choice is not unexpected as it is the most desired criterion for any breeder and farmer [30, 71].

Our result was contrary to Zavinon et al. [11] who found high market value to be the famers’ main preference criterion. In fact, the high market value cannot appear as the first preference criteria, because this criterion could only be the result of the adoption of an improved variety for one of the other criteria. Our study also revealed that preference criteria varied across different sociolinguistic groups, however, convergence in preference criteria between certain sociolinguistic groups was observed. This could be explained by the cultural links and the intensive knowledge exchange between these sociolinguistic groups or due to the common origin of the groups.

For a given character, a landrace does not show the same performance across the different ecological zones. For instance, the landrace called *Otili founfoun kékélé* (Idaasha sociolinguistic group) perceived by farmers as having high productivity in the Guinean and Sudano-Guinean zones showed low productivity in the Sudanian zone. This may be due to the variability in soil types, fertility, and organic matter turn over, soil nutrient dynamics [72], and water regime [73] across ecological zones. In addition, the landrace called *Klouékoun vôvô* (Fon and Mahi sociolinguistic groups) showed high productivity in the Guinean and Sudano-Guinean zones but low productivity in the Sudanian zone. Thus, as mentioned, variability in soil types, fertility and organic matter turn over, soil nutrient dynamics or water regime justify these agronomical differences.

*Carder ekloui* (Adja sociolinguistic group) only identified in the Guinean zone must deserve attention. The cultivar combined four good performances—high productivity, short cooking time, resistance to pests and diseases, resistance to storage insects—according to famers. *Carder ekloui* seems to be a promising cultivar which unfortunately faces threat of disappearance. There is urgent need to process to ex situ as well in situ conservation to preserve this cultivar and all those under threat. All identified cultivars in the current study must be tested to verify the performances stated by the farmers. Consequently, morphological and molecular characterizations are highly recommended to select suitable cultivars for breeding programs. Thereafter, association mapping of candidates’ genes/QTLs for desirables traits can be done and used in future marker-assisted breeding programs. Breeding of adapted pigeonpea resistant to pests and diseases and adaptable to any type of soil will be of dual benefit to famers in the Sudanian zone. It will enhance pigeonpea’s chain value and will also restore the fertilizing power of impoverished lands. Considering farmers’ preference criteria, the performing cultivars identified can be used in varietal exchange programmes to enhance pigeonpea production in Benin.

## Conclusion

Our study showed a diverse variety of pigeonpeas with a total of 15 landraces identified based on seed characteristics. Seven new landraces were found and a number of them were specific to an agro-ecological zone. A highly significant decrease in cropping areas was observed in the Guinean and Sudano-Guinean zones. Several factors including pests and diseases and long vegetative cycle constrain pigeonpea production. The absence of a performing seed system was also observed. Through participatory evaluation, this study revealed the existence of a few performing cultivars and cultivars under threat of disappearance. The establishment of an effective seed system and the definition of efficient pest management strategies, breeding, or introducing varieties based on farmers’ preference criteria could increase pigeonpea production in Benin. A few of the performing cultivars identified in the study can be used to mitigate the effects of identified constraints in varietal exchange programs. Morphological and molecular characterizations of identified cultivars are highly recommended to select suitable cultivars for breeding programs. In situ and ex situ conservation strategies and preservation of traditional knowledge associated to pigeonpea are essential to preserve landraces threatened to disappear and to conserve pigeonpea diversity in Benin.

## Data Availability

Raw and treated data generated during the study are available from the corresponding authors on reasonable request.

## Abbreviations

*p*: *p* value
INSAE: Institut National de la Statistique et de l’Analyse Economique
